# Combined associations of 25-hydroxivitamin D and parathyroid hormone with diabetes risk and associated comorbidities among U.S. white and black women

**DOI:** 10.1038/s41387-021-00171-2

**Published:** 2021-09-16

**Authors:** Jin Xia, Wanzhu Tu, JoAnn E. Manson, Hongmei Nan, Aladdin H. Shadyab, Jennifer W. Bea, Emily W. Gower, Lihong Qi, Ting-Yuan David Cheng, Yiqing Song

**Affiliations:** 1 Department of Epidemiology, Indiana University Richard M. Fairbanks School of Public Health, Indianapolis, IN USA; 2 Department of Biostatistics, Indiana University School of Medicine, Indianapolis, IN USA; 3 Division of Preventive Medicine, Department of Medicine, Brigham and Women’s Hospital, Harvard Medical School, Boston, MA USA; 4 Department of Epidemiology, Harvard T.H. Chan School of Public Health, Boston, MA USA; 5 Family Medicine and Public Health, School of Medicine, University of California, San Diego, La Jolla, CA USA; 6 University of Arizona Cancer Center, College of Medicine, Tucson, AZ USA; 7 Department of Epidemiology, Gillings School of Global Public Health, University of North Carolina, Chapel Hill, NC USA; 8 Department of Public Health, School of Medicine, University of California, Davis, CA USA; 9 Department of Epidemiology, College of Public Health and Health Professions, University of Florida, Gainesville, FL USA

**Keywords:** Cardiovascular diseases, Risk factors

## Abstract

**Background/objectives:**

There is evidence of black–white differences in vitamin D status and cardiometabolic health. This study aimed to further evaluate the joint associations of 25-hydroxyvitamin D [25(OH)D] and parathyroid hormone (PTH) with risks of diabetes and related cardiometabolic comorbidities among white and black women.

**Subjects/methods:**

We cross-sectionally and prospectively analyzed data from 1850 black and 3000 white postmenopausal women without cardiovascular disease or dialysis at baseline from the Women’s Health Initiative—Observational Study. Weighted Cox proportional hazards analyses and weighted logistic regression models were used to examine the joint associations of 25(OH)D and PTH with incident diabetes and prevalence of other diabetes-related cardiometabolic comorbidities (including CKD, hypertension, or obesity).

**Results:**

We identified 3322 cases of obesity (*n* = 1629), hypertension (*n* = 2759), or CKD (*n* = 318) at baseline and 453 incident cases of diabetes during 11 years of follow-up. Cross-sectionally, lower 25(OH)D and higher PTH were independently associated with higher prevalence of hypertension [odds ratio (OR) = 0.79; 95% confidence interval (CI): 0.72–0.87 and OR = 1.55; 95% CI: 1.39–1.73] among white women only. When stratified by diabetes status, compared to women with 25(OH)D ≥50 nmol/L and PTH ≤6.89 pmol/L (65 pg/mL), women who did not have diabetes with vitamin D deficiency (<50 nmol/L) and PTH excess (>6.89 pmol/L) had higher prevalence of CKD, hypertension, or obesity (OR = 4.23; 95% CI: 2.90–6.18) than women who had diabetes (OR = 1.89; 95% CI: 0.96–3.71). Prospectively, lower 25(OH)D was associated with lower diabetes incidence [hazard ratio (HR) = 0.73; 95% CI: 0.62–0.86] in white women. Jointly, compared to the group with 25(OH)D ≥50 nmol/L and PTH ≤6.89 pmol/L, white women with 25(OH)D deficiency (<50 nmol/L) had elevated risk for diabetes, regardless of PTH levels.

**Conclusions:**

Low 25(OH)D and high PTH were jointly associated with increased risk of diabetes among white women only. Their joint associations with high prevalence of CKD, hypertension, and obesity were more pronounced among women without diabetes.

## Introduction

There is consistent evidence that black Americans tend to have lower total 25-hydroxy vitamin D [25(OH)D] levels than white Americans, potentially due to reduced cutaneous biosynthesis of vitamin D [1, 2]. Greater cardiometabolic risk in blacks than whites has also been documented [3]. The vitamin D and parathyroid hormone (PTH) endocrine system plays a role in modulating a myriad of cardiometabolic risk factors through binding to the nuclear vitamin D receptor in a variety of tissues; regulatory targets include insulin/glucose metabolism, the renin–angiotensin–aldosterone system (RAAS), endothelial function, immune response modulation, cell differentiation and growth, and vascular and cardiac cell function [4–7]. Despite inconclusive results from randomized clinical trials regarding the effect of vitamin D supplementation on risk of diabetes and its metabolic risk factors [8, 9], data from observational studies have consistently shown associations between vitamin D deficiency and diabetes-related cardiometabolic disorders, such as obesity, hypertension, and chronic kidney disease (CKD) [10–12].

Emerging evidence also suggests the potential synergistic associations of vitamin D and PTH with cardiometabolic outcomes [13]. However, few studies have specifically examined the joint associations of circulating 25(OH)D and PTH levels with diabetes or related comorbid cardiometabolic conditions in a multiethnic cohort.

In the present study, using data from the Women’s Health Initiative—Observational Study (WHI-OS), we specifically examined (1) the joint associations of 25(OH)D and PTH with risk for developing diabetes among U.S. white and black postmenopausal women and (2) the joint associations of 25(OH)D and PTH with diabetes-related cardiometabolic comorbidities, including obesity, hypertension, and CKD, among women with diabetes compared to those without diabetes.

## Subjects and methods

### Study population

This study used data from an existing case–cohort study that is an ancillary study of the WHI-OS that enrolled 93,676 postmenopausal women aged 50–79 years from 40 clinical centers throughout the United States from 1994 to 1998 [14]. In the prospective case–cohort study for cardiovascular endpoints, 4850 women (1850 non-Hispanic black women and 3000 non-Hispanic white women; referred to hereafter as black women and white women, respectively) were selected after excluding those with history of stroke or myocardial infarction or who were receiving dialysis at baseline and followed up for an average of 11 years. Details of the WHI-OS cohort and the ancillary study design have been reported elsewhere [14].

Of the 4850 women, 622 women with diabetes and 4228 women without diabetes were identified at baseline. After removing 37 women with missing information on newly diagnosed diabetes, data on 4191 women were analyzed to examine the prospective associations between vitamin D biomarkers and incident diabetes among postmenopausal women (Supplementary Fig. 1). All participants provided written informed consent, and the study was approved by institutional review boards at each participating institution.

### Outcomes

#### Prevalent and incident diabetes

Prevalent diabetes was defined as a self-report of ever having received a physician diagnosis of diabetes when not pregnant or fasting plasma glucose levels of ≥6.99 mmol/L at baseline. Incident treated diabetes was defined as a self-report of a new physician diagnosis of diabetes treated with oral drugs or insulin shots during study follow-up, which has been confirmed to be consistent with medication inventories [15].

#### Diabetes-related cardiometabolic comorbidities

Obesity was defined as body mass index (BMI) ≥30 kg/m^2^, calculated based on clinical measurements of height and weight at baseline. Hypertension was defined as self-report of current treatment for hypertension and/or self-report of being told by a doctor they had high blood pressure and/or baseline measurement of systolic blood pressure ≥140 mmHg or diastolic blood pressure ≥90 mmHg. CKD was defined as an estimated glomerular filtration rate (eGFR) of <60 mL/min/1.73 m^2^ at study baseline. To achieve adequate statistical power, a composite endpoint of obesity, hypertension, or CKD was classified as no comorbidity or at least one comorbidity.

### Biomarker measurement

A 12-h fasting blood sample was collected at baseline from each participant and stored at −80 °C until assay. All biochemical assays were completed by Dr. Nader Rifai’s Clinical & Epidemiologic Research Laboratory. Plasma total 25(OH)D was assayed using an enzyme immunoassay with a competitive binding technique from Immunodiagnostic Systems Inc. (Fountain Hills, AZ, USA). PTH, creatinine, high-sensitivity C-reactive protein (hs-CRP), fasting glucose, and fasting insulin were measured by an electrochemiluminescence immunoassay on the Roche E Modular system using Roche Diagnostic reagents (Roche Diagnostics, Indianapolis, IN, USA). Mean intra-assay coefficients of variation for each analyte were 6.95% for 25(OH)D, 3.46% for PTH, 1.82% for creatinine, 3.34% for hs-CRP, 3.26% for fasting glucose, and 2.49% for fasting insulin.

eGFR was calculated using the Chronic Kidney Disease Epidemiology Collaboration equation, which provides more accurate prognostic information in the higher eGFR range than the Modification of Diet in Renal Disease equation [16].

eGFR (in ml/min/1.73 m^2^) = 141 × min(creatinine/*κ*, 1)^*α*^ × max(creatinine/*κ*, 1)^−1.209^ × 0.993^Age^ × 1.018 (if female) × 1.159 (if black), where creatinine = standardized serum creatinine measures in mg/dL; *κ* = 0.7 for females; *α* = −0.329 for females; min = indicates the minimum of creatinine/*κ* or 1; max = indicates the maximum of creatinine/*κ* or 1; Age = years.

### Covariates

Information on age (year), race/ethnicity (white or black), clinical center (Southern: <35°N, Middle: 35–40°N, and Northern: >40°N), education (≤high school graduate/General Educational Development, post high school, and college graduate or higher), season of blood draw (Spring, Summer, Autumn, and Winter), smoking status (never, past, and current), alcohol consumption (never, past, and current), physical activity levels (MET-hour/week), family history of diabetes, family history of cardiovascular disease (CVD; yes or no), history of high cholesterol (yes or no), postmenopausal hormone therapy use (never, past, and current), and statin use (yes or no) were obtained by self-report questionnaires from each participant at baseline. BMI (kg/m^2^) was calculated based on clinical measurements of height and weight.

### Statistical analysis

We compared baseline characteristics of white and black women according to status of vitamin D biomarkers using Kruskal–Wallis tests or Chi-square tests. Age-adjusted Spearman correlated coefficients were calculated to evaluate associations between vitamin D biomarkers and cardiometabolic biomarkers (i.e., creatinine, hs-CRP, fasting glucose, and insulin) among women without diabetes, stratified by race/ethnicity.

In the ancillary case–cohort study, a race-stratified probability sampling of the case and control subcohorts has been used to select the study samples of white and black women with different sampling fractions. To reflect the WHI-OS cohort characteristics, we estimated weights using the inverse of the sampling probability of the subcohort stratified by race based on the Barlow’s approach [17]. To evaluate the prospective associations between vitamin D biomarkers and incident diabetes, we fitted inverse-probability weighted Cox proportional-hazards models in women without diabetes at baseline. Follow-up time was calculated from enrollment to self-reported diabetes status at the annual visit, death, or to the last follow-up as of September 2013, whichever came first. In the analysis for independent associations, both baseline total 25(OH)D and PTH levels were modeled as continuous variables and quartiles. An interaction term between each vitamin D biomarker and race/ethnicity was added to test racial/ethnic differences in their independent associations. To assess their joint associations with diabetes, we also divided the sample into four subgroups by 25(OH)D (<50 vs. ≥50 nmol/L) and PTH (≤6.89vs. >6.89 pmol/L; equivalent to 65 pg/mL), with women having high 25(OH)D levels (>50 nmol/L) and low PTH levels (≤6.89 pmol/L) as the reference group. We further conducted a race/ethnicity-stratified analysis to examine the possible black–white differences in the associations between vitamin D biomarkers and incident diabetes. Our main models were adjusted for age, race/ethnicity, clinical center, BMI, education, season of blood draw, smoking status, alcohol consumption, physical activity levels, family history of diabetes, and postmenopausal hormone therapy use.

To take into account the sampling strategies (different subcohort sampling fractions were used for selecting controls and incident cases among white women and black women) used in the case–cohort study, we compared the weighted distributions of each vitamin D biomarker stratified by status of diabetes at baseline between white and black women, as well as between women with and without any diabetes-related cardiometabolic comorbidity. All statistics were calculated using the aforementioned inverse probability weighting method stratified by race. To cross-sectionally examine the independent and joint associations of vitamin D biomarkers with diabetes-related cardiometabolic comorbidities at baseline, weighted logistic regression models of women with and without prevalent or incident diabetes among all participants and each racial/ethnic group were constructed for each diabetes-related cardiometabolic comorbidity and the composite endpoint (CKD, hypertension, or obesity). Similar to the prospective analyses for incident diabetes, weights were estimated using the aforementioned race-stratified inverse probability sampling method to reflect the entire WHI-OS cohort. In the main models for prevalent obesity and the composite cardiometabolic endpoint, we controlled for age, race/ethnicity, clinical center, education, season of blood draw, smoking status, alcohol consumption, physical activity, and postmenopausal hormone therapy use. BMI was additionally adjusted in the analyses of prevalent CKD and hypertension. We also added interaction terms between race/ethnicity and each vitamin D biomarker into the main models to test for black–white differences. Multivariable weighted logistic regression models were also constructed to assess the joint associations of 25(OH)D and PTH with the composite endpoint in women with and without diabetes, separately.

To test the robustness of our results, we also performed several sensitivity analyses by further adjusting for eGFR (for incident diabetes and prevalence of obesity and hypertension only), history of high cholesterol, and statin use. All statistical analyses were performed using SAS version 9.4 (SAS Institute, Cary, NC), and a *P* value < 0.05 was considered statistically significant.

## Results

Table 1 depicts the characteristics of white and black women by the four subgroups of vitamin D biomarkers at baseline. Compared to women with non-deficient levels of 25(OH)D (≥50 nmol/L) and normal PTH levels (≤6.89 pmol/L), women with either deficient 25(OH)D levels (<50 nmol/L) or PTH excess (>6.89 pmol/L) had higher BMI, lower physical activity, and higher fasting glucose and insulin and prevalence of diabetes, obesity, and hypertension among both white and black women. In addition, women with PTH excess alone were younger and had lower eGFR, as well as higher creatinine levels and prevalence of CKD. Further, among subjects with vitamin D deficiency, white women were more likely than black women to have a family history of diabetes. With an average follow-up of 12.1 years, we identified 453 incident cases of treated diabetes among 4191 women without diabetes at baseline and 3322 prevalent cases of obesity (*n* = 1629), hypertension (*n* = 2759), or CKD (*n* = 318). The incidence rate of diabetes was 9.5 per 1000 person-years.

**Table 1:** Baseline characteristics stratified by race/ethnicity and vitamin D status [25(OH)D and PTH levels].

Variables		White women (*N* = 3000)					Black women (*N* = 1850)				
		25(OH)D ≥ 50 PTH ≤ 6.89 (*n* = 2107)	25(OH)D < 50 PTH ≤ 6.89 (*n* = 664)	25(OH)D ≥ 50 PTH > 6.89 (*n* = 103)	25(OH)D < 50 PTH > 6.89 (*n* = 91)	*P* value	25(OH)D ≥ 50 PTH ≤ 6.89 (*n* = 569)	25(OH)D < 50 PTH ≤ 6.89 (*n* = 1034)	25(OH)D ≥ 50 PTH > 6.89 (*n* = 39)	25(OH)D < 50 PTH > 6.89 (*n* = 195)	*P* value
Age (years)	Mean ± SD	65.4 ± 7.3	65.7 ± 7.6	68.0 ± 6.1	67.7 ± 6.6	1 × 10^-3^	63.1 ± 7.5	62.0 ± 7.3	67.8 ± 7.6	63.5 ± 7.5	<1 × 10^−4^
BMI (kg/m^2^)	Median (IQR)	25.5 (22.9, 29.0)	28.2 (24.4, 32.1)	27.1 (23.7, 31.4)	31.2 (27.2, 35.2)	<1 × 10^−4^	28.6 (25.4, 32.5)	29.9 (26.4, 34.6)	29.5 (26.9, 35.7)	33.3 (27.8, 38.4)	<1 × 10^−4^
Family history of CVD	*n* (%)	1111 (56.0%)	357 (56.1%)	53 (53.5%)	59 (67.8%)	0.17	236 (45.7%)	407 (43.5%)	15 (41.7%)	75 (43.4%)	0.86
Family history of diabetes	*n* (%)	594 (28.4%)	219 (33.1%)	25 (24.3%)	34 (37.4%)	0.03	270 (47.9%)	471 (45.9%)	18 (46.2%)	85 (10.1%)	0.27
History of high cholesterol	*n* (%)	320 (15.5%)	118 (18.1%)	21 (20.8%)	18 (20.0%)	0.19	74 (13.3%)	175 (17.3%)	7 (18.4%)	37 (19.6%)	0.11
Physical activity (MET-hour/week)	Median (IQR)	11.3 (4.5, 21.8)	5.6 (0.75, 12.5)	10.0 (3.5, 16.0)	4.8 (0, 10.3)	<1 × 10^−4^	9.5 (2.5, 19.8)	5.0 (0.5, 13.3)	3.5 (0.8, 8.8)	4.2 (0.8, 11.0)	<1 × 10^−4^
Smoking status	*n* (%)										
Never		997 (48.2%)	321 (49.0%)	47 (45.6%)	42 (46.7%)	0.03	286 (51.3%)	483 (47.5%)	22 (56.4%)	91 (47.2%)	0.02
Past		962 (46.5%)	275 (42.0%)	50 (48.5%)	40 (44.4%)		226 (40.5%)	394 (38.7%)	16 (41.0%)	74 (38.3%)	
Current		111 (5.4%)	59 (9.0%)	6 (5.8%)	8 (8.9%)		46 (8.2%)	141 (13.9%)	1 (2.6%)	28 (14.5%)	
Alcohol consumption	*n* (%)										
Never		199 (9.5%)	72 (10.9%)	15 (14.6%)	7 (7.7%)	0.01	94 (16.7%)	198 (19.4%)	8 (20.5%)	47 (24.2%)	0.22
Past		383 (18.4%)	134 (20.3%)	26 (25.2%)	29 (31.9%)		183 (32.5%)	341 (33.3%)	16 (41.0%)	64 (33.0%)	
Current		1504 (72.1%)	453 (68.7%)	62 (60.2%)	55 (60.4%)		287 (50.9%)	484 (47.3%)	15 (38.5%)	83 (42.8%)	
Hormone therapy use	*n* (%)										
Never use		777 (36.9%)	321 (48.3%)	37 (35.9%)	41 (45.1%)	<1 × 10^−4^	319 (56.2%)	644 (62.5%)	22 (56.4%)	119 (61.0%)	0.27
Past use		353 (16.8%)	111 (16.7%)	11 (10.7%)	17 (18.7%)		88 15.5(%)	129 (12.5%)	6 (15.4%)	22 (11.3%)	
Current use		975 (46.3%)	232 (34.9%)	55 (53.4%)	33 (36.3%)		161 (28.4%)	257 (25.0%)	11 (28.2%)	54 (27.7%)	
Statin use	*n* (%)	353 (16.8%)	123 (18.5%)	25 (24.3%)	22 (24.2%)	0.06	80 (14.1%)	148 (14.3%)	8 (20.5%)	46 (23.6%)	0.01
Educational levels	*n* (%)										
≤High school graduate/GED		437 (20.7%)	166 (25.0%)	27 (26.2%)	21 (23.1%)	0.09	135 (23.7%)	293 (28.3%)	15 (38.5%)	56 (28.7%)	0.01
Post high school		781 (37.1%)	244 (36.8%)	34 (33.0%)	41 (45.1%)		195 (34.3%)	404 (39.1%)	13 (33.3%)	75 (38.5%)	
College graduate or higher		889 (42.2%)	254 (38.3%)	42 (40.8%)	29 (31.9%)		239 (42.0%)	337 (32.6%)	11 (28.2%)	64 (32.8%)	
Geographical latitudes (clinical center)	*n* (%)										
Southern: <35°N		649 (30.8%)	154 (23.2%)	31 (30.1%)	18 (19.8%)	1 × 10^−3^	193 (33.9%)	342 (33.1%)	14 (35.9%)	59 (30.3%)	0.22
Middle: 35–40°N		602 (28.6%)	197 (29.7%)	33 (32.0%)	24 (26.4%)		210 (36.9%)	329 (31.8%)	12 (30.8%)	73 (37.4%)	
Northern: >40°N		856 (40.6%)	313 (47.1%)	39 (37.9%)	49 (53.9%)		166 (29.2%)	363 (35.1%)	13 (33.3%)	63 (32.3%)	
Season of blood draw	*n* (%)										
Spring		550 (26.2%)	254 (38.4%)	30 (29.1%)	31 (34.1%)	<1 × 10^−4^	132 (23.4%)	320 (31.3%)	10 (25.6%)	65 (33.5%)	<1 × 10^−4^
Summer		658 (31.3%)	139 (21.0%)	32 (31.1%)	19 (20.9%)		176 (31.2%)	259 (25.3%)	12 (30.8%)	42 (21.7%)	
Autumn		503 (23.9%)	96 (14.5%)	26 (25.2%)	18 (19.8%)		161 (28.6%)	191 (18.7%)	8 (20.5%)	40 (20.6%)	
Winter		390 (18.6%)	172 (26.0%)	15 (14.6%)	23 (25.3%)		95 (16.8%)	253 (24.7%)	9 (23.1%)	47 (24.2%)	
Vitamin D biomarkers											
Total 25(OH)D, nmol/L	Median (IQR)	68.7 (59.2, 80.1)	41.8 (36.2, 46.4)	61.1 (55.9, 69.0)	38.1 (30.5, 42.8)	<1 × 10^−4^	61.7 (54.7, 70.5)	37.2 (31.4, 43.1)	57.7 (53.1, 63.5)	32.1 (26.2, 38.2)	<1 × 10^−4^
PTH, pmol/L	Median (IQR)	3.7 (3.0, 4.5)	4.4 (3.5, 5.2)	7.7 (7.2, 8.4)	8.2 (7.4, 10.0)	<1 × 10^−4^	3.7 (3.0, 4.6)	4.3 (3.5, 5.2)	8.0 (4.9, 6.4)	8.2 (7.5, 9.7)	<1 × 10^−4^
Cardiometabolic biomarkers											
Creatinine, µmol/L	Median (IQR)	63.6 (57.5, 71.6)	61.9 (55.7, 70.7)	67.2 (57.5, 82.2)	67.2 (59.2, 80.4)	<1 × 10^−4^	70.7 (61.0, 80.4)	68.1 (60.1, 76.9)	75.1 (61.9, 90.2)	74.3 (64.5, 91.1)	<1 × 10^−4^
hs-CRP, mg/L	Median (IQR)	2.4 (1.1, 5.4)	2.7 (1.3, 6.7)	4.0 (1.5, 9.1)	4.7 (1.9, 9.1)	<1 × 10^−4^	3.8 (1.5, 6.9)	3.4 (1.5, 8.0)	3.2 (2.3, 8.8)	4.4 (1.9, 10.1)	0.13
Glucose, mmol/L	Median (IQR)	5.2 (4.9, 5.6)	5.3 (4.9, 5.9)	5.2 (4.9, 5.7)	5.6 (5.1, 6.4)	<1 × 10^−4^	5.2 (4.8, 5.8)	5.3 (4.9, 6.0)	5.5 (4.9, 6.4)	5.3 (4.9, 6.0)	0.04
Insulin, pmol/L	Median (IQR)	46.3 (32.4, 70.0)	60.5 (39.2, 97.9)	53.8 (41.9, 80.9)	77.1 (50.1, 124.4)	<1 × 10^−4^	58.3 (38.5, 87.8)	64.0 (40.0, 98.7)	71.0 (39.1, 113.1)	76.0 (46.6, 120.1)	2 × 10^−4^
eGFR, mL/min/1.73 m^2^	Mean ± SD	84.0 ± 13.4	85.1 ± 13.6	75.2 ± 19.9	77.1 ± 18.8	<1 × 10^−4^	90.9 ± 17.9	94.4 ± 18.4	80.9 ± 25.6	83.2 ± 24.1	<1 × 10^−4^
Diabetes	*n* (%)	124 (5.9%)	92 (13.9%)	9 (8.7%)	19 (20.9%)	<1 × 10^−4^	104 (18.3%)	215 (20.8%)	8 (20.5%)	37 (19.0%)	0.67
Diabetes-related comorbidities											
Obesity	*n* (%)	410 (19.7%)	241 (36.5%)	33 (32.4%)	51 (58.6%)	<1 × 10^−4^	229 (41.0%)	504 (49.8%)	18 (46.2%)	121 (63.4%)	<1 × 10^−4^
Hypertension	*n* (%)	978 (46.7%)	389 (58.8%)	64 (62.1%)	65 (72.2%)	<1 × 10^−4^	367 (64.5%)	690 (66.7%)	33 (84.6%)	143 (73.3%)	0.01
Chronic kidney disease	*n* (%)	123 (5.8%)	35 (5.3%)	23 (22.3%)	15 (16.5%)	<1 × 10^−4^	30 (5.3%)	49 (4.7%)	7 (18.0%)	35 (18.0%)	<1 × 10^−4^

*P* values for comparisons by status of vitamin D biomarkers were obtained by Kruskal–Wallis tests for continuous variables and Chi-square tests for categorical variables.

*BMI* body mass index, *CVD* cardiovascular disease, *eGFR* estimated glomerular filtration rate, *GED* General Educational Development, *hs-CRP* high-sensitivity C-reactive protein, *25(OH)D* 25-hydroxyvitamin D, *MET* metabolic equivalent of task, *PTH* parathyroid hormone.

As expected, total 25(OH)D and PTH were significantly and inversely correlated across racial/ethnic groups among all women without diabetes (Table 2). Also, creatinine, hs-CRP, fasting glucose, and insulin were inversely correlated with 25(OH)D and positively correlated with PTH. After stratifying by race/ethnicity, the correlation between 25(OH)D and creatinine turned from negative to positive, while all others persisted among white women. Among black women, only the inverse correlation between 25(OH)D and fasting insulin remained significant. The positive correlations between PTH and the abovementioned CVD biomarkers were consistent across the two racial/ethnic groups.

**Table 2:** Correlations between vitamin D biomarkers and cardiometabolic biomarkers among non-diabetic women stratified by race/ethnicity.

Biomarkers	Total 25(OH)D	PTH	Creatinine	hs-CRP	Glucose	Insulin
All non-diabetic women (*n* = 4228)						
Total 25(OH)D	1	−0.36**	−0.07**	−0.09**	−0.08**	−0.2**
PTH		1	0.09**	0.08**	0.09**	0.15**
Creatinine			1	0.02	−0.01	0.09**
hs-CRP				1	0.11**	0.32**
Glucose					1	0.43**
Insulin						1
American white non-diabetic women (*n* = 2748)						
Total 25(OH)D	1	−0.3**	0.06**	−0.06**	−0.14**	−0.2**
PTH		1	0.05*	0.06**	0.1**	0.12**
Creatinine			1	−0.01	−0.02	0.05**
hs-CRP				1	0.08**	0.29**
Glucose					1	0.44**
Insulin						1
American black non-diabetic women (*n* = 1480)						
Total 25(OH)D	1	−0.36**	0.02	−0.03	−0.04	−0.1**
PTH		1	0.07**	0.07**	0.1**	0.16**
Creatinine			1	0.01	0.02	0.07**
hs-CRP				1	0.16**	0.34**
Glucose					1	0.41**
Insulin						1

Age-adjusted Spearman’s correlation coefficients are shown.

*P < 0.05; **P < 0.01.

**Fig. 1:**
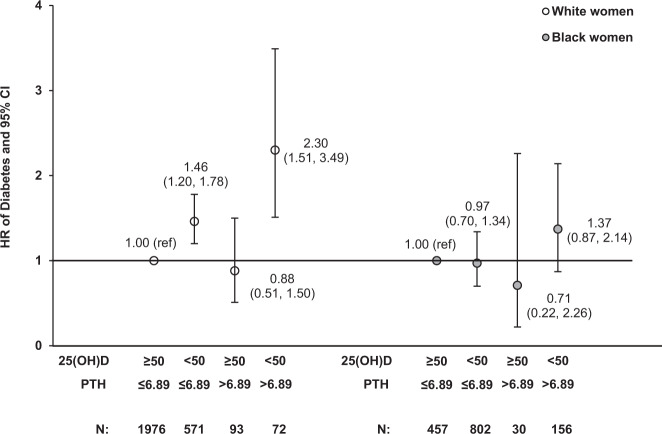
Joint associations of 25(OH)D and PTH with incident diabetes. HRs (95% CIs) of diabetes among U.S. white and black women were estimated. Models were adjusted for age, clinical center, race/ethnicity, BMI, family history of diabetes, educational levels, alcohol intake, physical activity levels, cigarette smoking status, postmenopausal hormone therapy use, and season of blood draw. *N* total number of participants.

In the multivariable-adjusted analysis, as expected, we found that higher 25(OH)D levels were significantly and independently associated with lower risk for incident diabetes in a dose-response manner among all participants [hazard ratio (HR) = 0.78 per-SD change in 25(OH)D, 95% confidence interval (CI): 0.68–0.88; *P* for linear trend <0.0001] and among white women (HR = 0.73 per-SD change in 25(OH)D, 95% CI: 0.62–0.86; *P* for linear trend <0.0001). However, we observed no significant association between PTH and diabetes in the fully adjusted model (Supplementary Table 1). Further, the interactions between vitamin D biomarkers and race/ethnicity were also not statistically significant. When assessed jointly, compared to those with both non-deficient 25(OH)D (≥50 nmol/L) and normal PTH (≤6.89 pmol/L), having deficient 25(OH)D levels (<50 nmol/L) was associated with significantly higher risk for diabetes, regardless of whether they had excess PTH levels (HR = 1.92; 95% CI: 1.42–2.58) or normal PTH levels (HR = 1.32; 95% CI: 1.12–1.57). The observed joint association of vitamin D deficiency and PTH excess with incident diabetes remained significant and became even stronger in white women (Fig. 1). White women with deficient 25(OH)D (<50 nmol/L) had higher risk of diabetes, regardless of their PTH level (HR = 1.46; 95% CI: 1.20–1.78 for those with normal PTH; HR = 2.30; 95% CI: 1.51–3.49 for those with excess PTH). Based on the HR being 1.46 for the group with vitamin D deficiency alone and 0.88 for the group with PTH excess only, the excess relative risk (ERR) associated with deficient 25(OH)D levels was calculated as 1.46 − 1 = 0.46 and the ERR related to PTH excess was −0.12, separately. The joint association of deficient 25(OH)D and excess PTH levels with incident diabetes (HR = 2.30) showed a trend toward synergism on an additive scale [relative excess risk due to interaction = 1.30 − 0.46 − (−0.02) = 0.86] among white women but did not reach statistical significance. However, there was no evidence of such an association among black women.

**Fig. 2:**
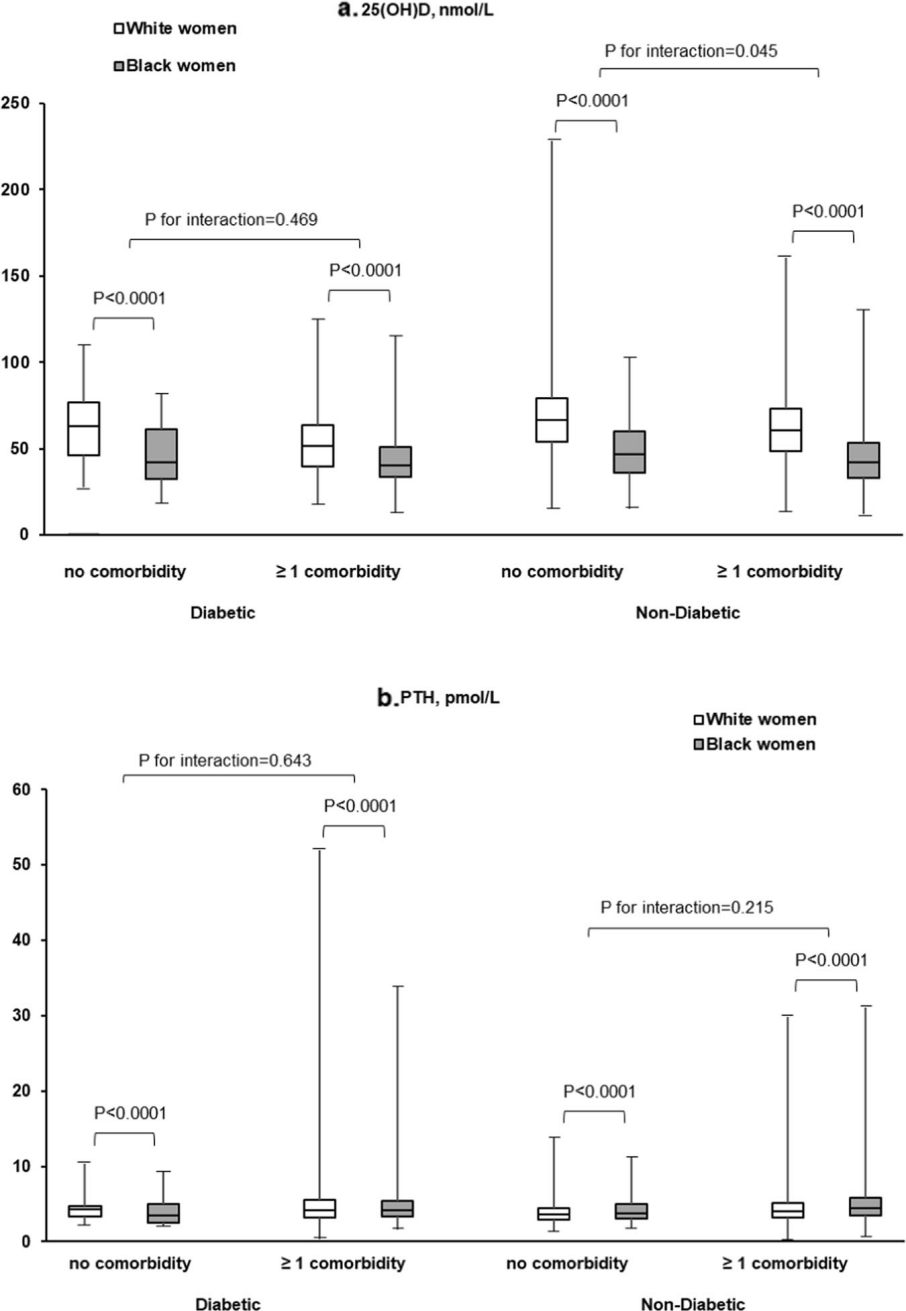
Distributions of vitamin D biomarkers at baseline. 25(OH)D (**a**) and PTH (**b**) by diabetes status at baseline and diabetes-related cardiometabolic comorbidities among U.S. white and black women. All *P* values were obtained from weighted regression models with an interaction term between race and comorbidity status.

Weighted distributions of 25(OH)D and PTH differed significantly between white and black women when stratified by diabetes status and diabetes-related cardiometabolic comorbidities (all *P* values < 0.0001; Fig. 2). We also observed that the interaction between race/ethnicity and diabetes-related cardiometabolic comorbidities was statistically significant for 25(OH)D (*P* for interaction = 0.045) among women without diabetes at baseline.

**Fig. 3:**
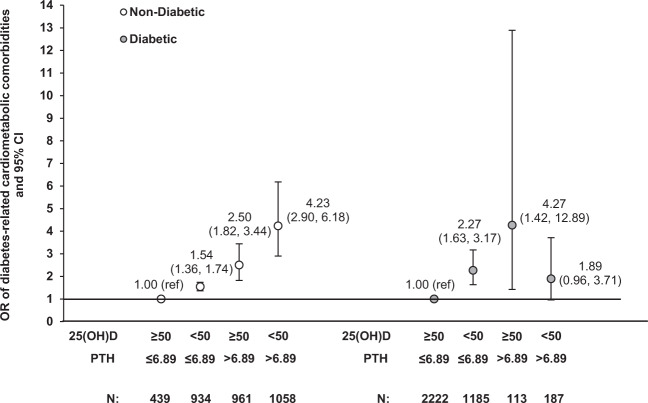
Joint associations of 25(OH)D and PTH with prevalence of diabetes-related cardiometabolic comorbidities. ORs (95% CIs) of CKD, hypertension, or obesity between women with and without prevalent or incident diabetes were estimated. Models were adjusted for age, clinical center, race/ethnicity, family history of CVD, educational levels, alcohol intake, physical activity levels, cigarette smoking status, postmenopausal hormone therapy use, and season of blood draw. *N* total number of participants.

Individually, lower 25(OH)D or higher PTH level was associated with greater prevalence of obesity and the composite endpoint across racial/ethnic groups, whereas their independent associations with hypertension were statistically significant in white women only (Supplementary Tables 2 and 3). In addition, we found a positive relationship between PTH and CKD among both white and black women (Supplementary Table 3). These results were not modified by additional adjustment for history of high cholesterol, statin use, and eGFR (for obesity and hypertension only). The interaction term between 25(OH)D and race/ethnicity was significant for obesity (*P* for interaction = 0.007; Supplementary Table 2). There was also a significant interaction between PTH and race/ethnicity on hypertension (*P* for interaction = 0.008; Supplementary Table 3). When 25(OH)D and PTH were assessed jointly (Fig. 3), women with either deficient 25(OH)D (<50 nmol/L) alone or excess PTH levels (>6.89 pmol/L) alone had higher prevalence of obesity, hypertension, or CKD, compared to those with non-deficient 25(OH)D (≥50 nmol/L) and normal PTH levels (≤6.89 pmol/L), regardless of diabetes status. However, the combination of deficient 25(OH)D levels and PTH excess was associated with higher prevalence for at least one cardiometabolic outcome among women without prevalent or incident diabetes [odds ratio (OR) = 4.23, 95% CI: 2.90–6.18] but not for those who had diabetes at baseline or who developed diabetes later (OR = 1.89, 95% CI: 0.96–3.71).

## Discussion

Among a cohort of U.S. black and white postmenopausal women, deficient 25(OH)D levels (<50 nmol/L) were significantly associated with increased risk of diabetes in white women only, regardless of PTH levels. Compared to those with non-deficient levels of 25(OH)D (≥50 nmol/L) and normal PTH levels (≤6.89 pmol/L), women with either deficient 25(OH)D or excess PTH levels had higher prevalence of cardiometabolic traits including obesity, hypertension, or CKD, irrespective of diabetes status; however, the combination of vitamin D deficiency and PTH excess was associated with higher prevalence of at least one of the aforementioned diabetes-related cardiometabolic comorbidities among women without diabetes only.

### Vitamin D and PTH with incident diabetes

Our findings agree with the results of two recent prospective population-based cohort studies and our previous meta-analysis of observational studies that showed independent associations between deficient vitamin D levels and diabetes [18–20]. Data from a U.S. cohort of 903 Caucasian adults aged 74 years on average and free of diabetes or pre-diabetes at study entry through a 12.5-year follow-up period suggested that 25(OH)D was inversely associated with incidence of diabetes in a dose-response manner, after adjusting for age, BMI, waist circumference, calcium supplement intake, triglycerides, and high-density lipoprotein-cholesterol [18]. A German population study of 7791 initially diabetes-free subjects aged 50–74 years reported a non-linear inverse relationship between 25(OH)D and risk of diabetes with a threshold of <40 nmol/L in women only after an 8-year follow-up, accounting for age, sex, season of blood draw, multi-vitamin supplement intake, frequent fish consumption, BMI, hemoglobin A1C, family history of diabetes, education, physical activity, smoking, hypertension, renal dysfunction, C-reactive protein, and fasting triglycerides [19]. A recent randomized clinical trial in Canada among 96 individuals with vitamin D insufficiency [mean 25(OH)D = 51.1 nmol/L at baseline] and at high risk of diabetes or with newly diagnosed type 2 diabetes found that vitamin D3 supplementation (5000 IU daily) for 6 months significantly increased peripheral insulin sensitivity and β-cell function [9].

Data on joint associations between vitamin D and PTH with diabetes and related risk factors are limited. In line with our findings, a prospective study of 494 postpartum women in Canada suggested that vitamin D deficiency/insufficiency with PTH in the highest tertile at 3 months postpartum was associated with worsening β-cell function and insulin sensitivity and increased fasting and 2-h glucose 9 months later, after controlling for age, ethnicity, family history of type 2 diabetes, previous gestational diabetes, BMI, glucose, duration of breast-feeding, physical activity, and season of blood draw [21]. Another case–control study in a Greek cohort of 144 patients aged ≥65 years with pre-diabetes and 81 healthy age-matched controls reported that individuals with 25(OH)D <50 nmol/L and PTH ≥40 pg/mL had significantly higher fasting plasma glucose levels than those with either 25(OH)D ≥50 nmol/L or PTH <40 pg/mL or both, after adjustment for age, sex, BMI, and season of sampling [22].

However, conflicting findings have also been reported. An Italian observational study in a cohort of 2227 elderly Caucasians aged 76.1 years on average age found no association between baseline 25(OH)D and incidence of diabetes over 4.4 years follow-up, potentially due to competing risk of death (22.4% lost to follow-up due to death) and a likely underestimation of newly diagnosed diabetes because of relying solely on fasting glucose [23]. Similarly, a recent large randomized clinical trial (*n* = 2423) indicated that intake of 4000 IU daily of vitamin D3 by participants with pre-diabetes did not significantly lower risk of diabetes onset through a median follow-up of 2.5 years [8]. The null findings may be due mainly to adequate vitamin D status at baseline of the trial participants with mean 25(OH)D levels of 70.5 nmol/L at baseline, which may have limited the ability of those researchers to detect a significant effect. Recently, our meta-analysis of 47 randomized controlled trials involving 44,161 individuals without diabetes found no effect of vitamin D supplementation on incidence of type 2 diabetes, but most trials were very small and included populations with varying levels of 25(OH)D at baseline (13.6–61.2 nmol/L) [24]. Our overall dose-response analyses suggested that vitamin D supplementation with a dose >4000 IU/day may be sufficient to achieve optimal 25(OH)D levels (>90 nmol/L) and improve glucose and insulin homeostasis [24].

### Vitamin D and PTH with diabetes-related cardiometabolic comorbidities

Similar to our findings for hypertension in white women, data from the third National Health and Nutrition Examination Survey indicated an inverse relationship between 25(OH)D and systolic blood pressure among white Americans [25]. Conversely, a cross-sectional study in 1205 adults aged ≥65 years in the Netherlands (predominantly Caucasians) reported that higher PTH levels, unlike 25(OH)D, were associated with risk of hypertension with adjustments for age, sex, region, season, waist circumference, physical activity, smoking, and alcohol intake [26]. Supporting our findings of an independent, positive association of PTH with CKD but not with 25(OH)D, the results from another recent cross-sectional study in a community population of 4080 adults in Taiwan suggested that either elevated PTH levels or hyperparathyroidism was associated with higher risk of CKD after adjusting for age, sex, 25(OH)D, calcium, and phosphate [27]. In contrast, an inverse association between 25(OH)D and risk of incident CKD was identified in a Chinese elderly cohort (*n* = 1037), accounting for potential confounders [28]. While evidence suggests that obesity is inversely associated with 25(OH)D and positively associated with PTH [29], conflicting results also exist [30]. A retrospective study of 316 patients in a Spanish hospital found that neither 25(OH)D nor PTH was associated with obesity after adjusting for potential confounding factors [30].

The combined associations we observed between vitamin D deficiency and PTH excess with at least one cardiometabolic condition (i.e., obesity, hypertension, or CKD) and their joint association with CVD risk in women without diabetes imply that the combined assessment of vitamin D and PTH may more accurately reflect the functional status of vitamin D relevant to cardiometabolic health compared with assessment of one factor alone. Our results also indicate that the combined associations of deficient vitamin D and elevated PTH with the aforementioned cardiometabolic conditions may vary depending on diabetes status. Our estimated partial Spearman’s correlations show that creatinine and hs-CRP, as well as fasting glucose and insulin, were inversely correlated with 25(OH)D and positively correlated with PTH among individuals without diabetes. Among women with diabetes, we found that 25(OH)D was still inversely and significantly associated with fasting glucose but not with fasting insulin, while PTH was not associated with either fasting glucose or fasting insulin in white women with diabetes. Overall, our findings may help explain inconsistent results regarding the associations of vitamin D and PTH with cardiometabolic conditions and thus inform the design of future observational and interventional studies assessing the impact of the vitamin D–PTH endocrine system on cardiometabolic health.

### Potential mechanisms

The mechanisms underlying racial differences in the vitamin D–PTH axis and their contributions to health outcomes are complex and remain elusive. It is well documented that RAAS activation is associated with hypertension, CKD, diabetes, and CVD through its crucial role in the regulation of blood pressure, fluid volume, and electrolyte homeostasis [31, 32]. Blood pressure sensitivity to aldosterone appears to be population specific, e.g., blacks on average have lower aldosterone levels in the circulation [33]. Mechanistic studies in both animals and humans have shown vitamin D to be a negative endocrine regulator of the RAAS [4, 34]. Vitamin D deficiency, which is more common in blacks, tends to upregulate the RAAS by increasing renin and aldosterone production [34]. A small increase in aldosterone can increase blood pressure, more so in blacks than in whites. PTH excess itself may stimulate adrenal aldosterone synthesis directly via binding to PTH receptors and, in concert with vitamin D, may indirectly stimulate renin release to increase angiotensin II [35]. Other possible mechanisms underlying the complex interplay between vitamin D, PTH, and cardiometabolic disorders include stimulation of lipogenesis and insulin secretion, inhibition of lipolysis and pro-inflammatory cytokine production, and modulation of endothelial functions, through the expression of vitamin D and PTH receptors in adipocytes, vascular smooth muscle cells, and endothelial cells [5, 36–41]. There is compelling evidence demonstrating race/ethnicity-specific thresholds for defining vitamin D deficiency [14, 42]. Previous studies have also documented consistent and substantial white–black differences in PTH response to 25(OH)D levels. Taken together, collective evidence supports physiological differences between whites and blacks in altered vitamin D/PTH endocrine system and may explain at least in part the racial differences in hypertension and other cardiometabolic disorders associated with 25(OH)D and PTH.

### Strengths and limitations

Our study has several strengths. The large and well-characterized cohort of white and black postmenopausal women with a long follow-up allowed us to address racial/ethnic differences in the associations of total 25(OH)D and PTH with cardiometabolic risk. We were also able to examine multiple established diabetes-related cardiometabolic comorbidities, accounting for many potential confounders. However, our cross-sectional study design did not allow us to establish a temporal relationship between total 25(OH)D/PTH and development of these comorbidities or other diabetes-related cardiometabolic comorbid conditions. Also, random measurement error from single measurement of biomarkers might have biased our results toward the null. However, this seems unlikely, since all the coefficients of variation were <10% and were similar to those reported in the majority of previous population studies. In addition, residual or unmeasured confounding cannot be ruled out, such as that derived from sun exposure and dietary or supplemental vitamin D intake. Lastly, we are unable to generalize our results from postmenopausal women to men or other populations.

## Conclusions

This prospective population-based cohort study suggests a joint association of vitamin D deficiency and excess PTH with risk of developing diabetes among U.S. white postmenopausal women. Either vitamin D deficiency or excess PTH was significantly associated with the prevalence of obesity, hypertension, or CKD, irrespective of diabetes status. Larger longitudinal studies are needed to confirm our results and to determine race-specific thresholds of both 25(OH)D and PTH concentrations and their trajectories in relation to future risk of cardiometabolic diseases. If confirmed, future large-scale randomized controlled trials among participants with true vitamin D deficiency are warranted to clarify the preventive dosage of vitamin D supplementation and to address racial differences of CVD risk caused by vitamin D/PTH endocrine dysfunction.

## Supplementary information

Supplementary Figure 1

Supplemental Table 1

Supplemental Table 2

Supplemental Table 3
