# Supplementary figures and images for: Combined associations of 25-hydroxivitamin D and parathyroid hormone with diabetes risk and associated comorbidities among U.S. white and black women
Source: Nutr Diabetes. 2021 Sep 16;11:29. doi: 10.1038/s41387-021-00171-2 (PMC8676147; doi:10.1038/s41387-021-00171-2)

## Supplementary Tables and Figures

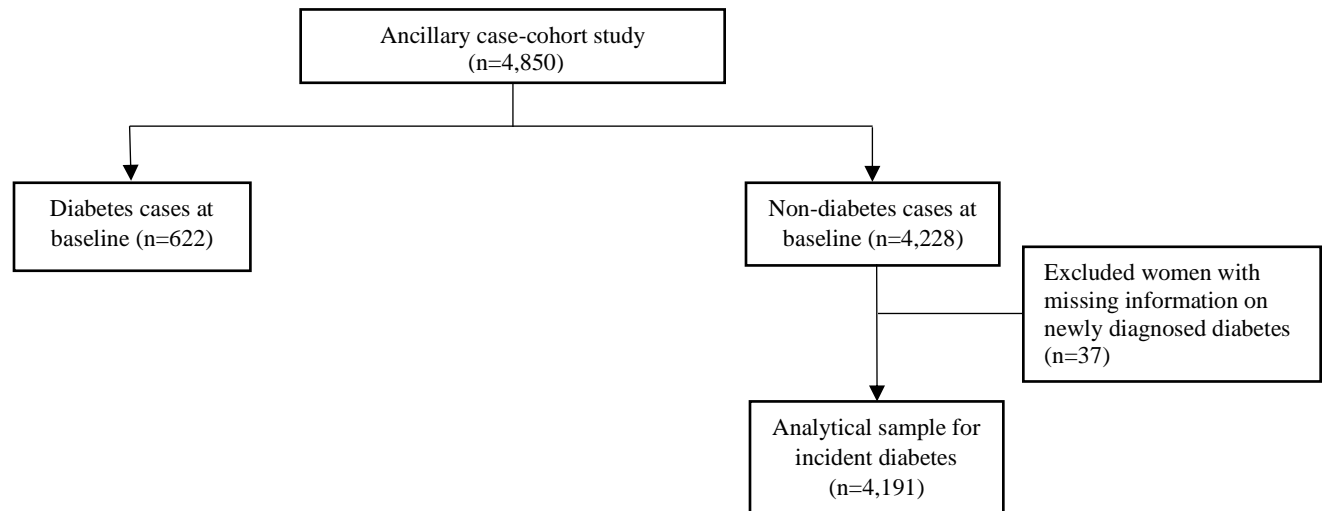

**Supplemental Fig. 1.** Participant flow diagram of study design.

Supplement: Supplementary file 1 — Supplementary Figure 1 [file 41387_2021_171_MOESM1_ESM.pdf]
