# Supplementary material for: Combined associations of 25-hydroxivitamin D and parathyroid hormone with diabetes risk and associated comorbidities among U.S. white and black women
Source: Nutr Diabetes. 2021 Sep 16;11:29. doi: 10.1038/s41387-021-00171-2 (PMC8676147; doi:10.1038/s41387-021-00171-2)
Supplement: Supplementary file 2 — Supplemental Table 1 [file 41387_2021_171_MOESM2_ESM.docx]

**Supplemental Table 1.** Independent associations of 25(OH)D and PTH with incidence of diabetes.

| **Model** | | **HR (95%CI)** | | | | ***P* for linear trend** | **HR _per-SD_**^a^ **(95%CI)** | ***P* for interaction**^b^ |
| --- | --- | --- | --- | --- | --- | --- | --- | --- |
|  |  | **Quartile 1** | **Quartile 2** | **Quartile 3** | **Quartile 4** |  |  |  |
| **Total 25(OH)D** | |  |  |  |  |  |  |  |
| All participants | Model 1^c^ | 1 | 0.63 (0.53, 0.76) | 0.53 (0.44, 0.64) | 0.42 (0.34, 0.52) | < 0.0001 | 0.63 (0.56, 0.71) | 0.659 |
|  | Model 2^d^ | 1 | 0.76 (0.63, 0.92) | 0.69 (0.56, 0.85) | 0.57 (0.45, 0.72) | < 0.0001 | 0.78 (0.68, 0.88) | 0.313 |
|  | Model 3^e^ | 1 | 0.76 (0.63, 0.92) | 0.67 (0.54, 0.83) | 0.58 (0.45, 0.73) | < 0.0001 | 0.78 (0.68, 0.89) | 0.254 |
|  |  |  |  |  |  |  |  |  |
| American white women | Model 1^c^ | 1 | 0.60 (0.48, 0.74) | 0.6 (0.49, 0.74) | 0.44 (0.35, 0.55) | < 0.0001 | 0.60 (0.52, 0.69) | n/a |
|  | Model 2^d^ | 1 | 0.66 (0.53, 0.82) | 0.75 (0.60, 0.94) | 0.55 (0.42, 0.71) | < 0.0001 | 0.73 (0.62, 0.86) | n/a |
|  | Model 3^e^ | 1 | 0.64 (0.51, 0.80) | 0.70 (0.56, 0.89) | 0.57 (0.44, 0.73) | < 0.0001 | 0.73 (0.62, 0.86) | n/a |
|  |  |  |  |  |  |  |  |  |
| American black women | Model 1^c^ | 1 | 0.86 (0.62, 1.21) | 0.74 (0.52, 1.05) | 0.48 (0.32, 0.71) | 0.0001 | 0.80 (0.69, 0.94) | n/a |
|  | Model 2^d^ | 1 | 0.96 (0.67, 1.38) | 0.98 (0.67, 1.43) | 0.65 (0.42, 0.997) | 0.052 | 0.93 (0.79, 1.10) | n/a |
|  | Model 3^e^ | 1 | 0.96 (0.67, 1.39) | 1.01 (0.69, 1.47) | 0.64 (0.42, 0.99) | 0.051 | 0.94 (0.79, 1.11) | n/a |
|  |  |  |  |  |  |  |  |  |
| **PTH** |  |  |  |  |  |  |  |  |
| All participants | Model 1^c^ | 1 | 1.04 (0.85, 1.26) | 0.87 (0.71, 1.07) | 1.45 (1.20, 1.74) | < 0.0001 | 1.17 (1.08, 1.27) | 0.688 |
|  | Model 2^d^ | 1 | 0.94 (0.77, 1.15) | 0.78 (0.63, 0.96) | 1.13 (0.93, 1.37) | 0.152 | 1.05 (0.96, 1.16) | 0.905 |
|  | Model 3^e^ | 1 | 0.95 (0.77, 1.16) | 0.78 (0.63, 0.97) | 1.11 (0.91, 1.35) | 0.215 | 1.04 (0.94, 1.15) | 0.943 |
|  |  |  |  |  |  |  |  |  |
| American white women | Model 1^c^ | 1 | 1.25 (1.00, 1.57) | 0.92 (0.72, 1.18) | 1.39 (1.11, 1.74) | 0.018 | 1.16 (1.04, 1.29) | n/a |
|  | Model 2^d^ | 1 | 1.10 (0.87, 1.39) | 0.83 (0.64, 1.06) | 1.16 (0.92, 1.46) | 0.366 | 1.05 (0.93, 1.18) | n/a |
|  | Model 3^e^ | 1 | 1.08 (0.85, 1.37) | 0.80 (0.62, 1.03) | 1.11 (0.87, 1.40) | 0.577 | 1.02 (0.90, 1.15) | n/a |
|  |  |  |  |  |  |  |  |  |
| American black women | Model 1^c^ | 1 | 0.77 (0.53, 1.13) | 0.85 (0.59, 1.24) | 1.39 (0.99, 1.94) | 0.016 | 1.17 (1.03, 1.34) | n/a |
|  | Model 2^d^ | 1 | 0.68 (0.46, 1.01) | 0.62 (0.41, 0.93) | 0.99 (0.69, 1.43) | 0.616 | 1.04 (0.89, 1.23) | n/a |
|  | Model 3^e^ | 1 | 0.70 (0.47, 1.03) | 0.64 (0.43, 0.97) | 0.99 (0.68, 1.43) | 0.63 | 1.05 (0.89, 1.24) | n/a |

^a^ HRs represent per-standard deviation increases in biomarker measures.

^b^ P for interaction was obtained by adding an interaction term between each vitamin D biomarker and race/ethnicity into each model; n/a indicates that interaction tests for race/ethnicity were not applicable.

^c^ Model 1 adjusted for age, clinical center, and race/ethnicity.

^d^ Model 2 further adjusted for BMI, family history of diabetes, educational levels, alcohol intake, physical activity levels, cigarette smoking status, postmenopausal hormone therapy use, and season of blood draw.

^e^ Model 3 additionally adjusted for eGFR, history of high cholesterol and statin use.
