# Supplementary material for: Combined associations of 25-hydroxivitamin D and parathyroid hormone with diabetes risk and associated comorbidities among U.S. white and black women
Source: Nutr Diabetes. 2021 Sep 16;11:29. doi: 10.1038/s41387-021-00171-2 (PMC8676147; doi:10.1038/s41387-021-00171-2)
Supplement: Supplementary file 4 — Supplemental Table 3 [file 41387_2021_171_MOESM4_ESM.docx]

**Supplemental Table 3.** Independent associations of PTH with prevalence of comorbidities (obesity, hypertension, or CKD).

| **Model** | | **OR (95%CI)** | | | | | | | ***P* for linear trend** | | **OR _per-SD_**‡ **(95%CI)** | | ***P* for interaction**§ | |
| --- | --- | --- | --- | --- | --- | --- | --- | --- | --- | --- | --- | --- | --- | --- |
|  |  | **Quartile 1** | **Quartile 2** | | **Quartile 3** | | **Quartile 4** | |  |  |  |  |  |  |
| **Obesity** |  |  |  | |  | |  | |  | |  | |  | |
| All participants | Model 1^c^ | 1 | 1.30 (1.14, 1.49) | | 1.44 (1.26, 1.65) | | 2.49 (2.18, 2.83) | | < 0.0001 | | 1.75 (1.61, 1.89) | | 0.595 | |
|  | Model 2^d^ | 1 | 1.25 (1.08, 1.44) | | 1.39 (1.20, 1.61) | | 2.10 (1.82, 2.41) | | < 0.0001 | | 1.58 (1.45, 1.72) | | 0.949 | |
|  | Model 3^e^ | 1 | 1.25 (1.08, 1.44) | | 1.38 (1.19, 1.59) | | 2.10 (1.82, 2.41) | | < 0.0001 | | 1.58 (1.45, 1.72) | | 0.949 | |
|  |  |  |  | |  | |  | |  | |  | |  | |
| American white women | Model 1^c^ | 1 | 1.55 (1.32, 1.81) | | 1.39 (1.19, 1.63) | | 2.52 (2.17, 2.93) | | < 0.0001 | | 1.86 (1.68, 2.06) | | n/a | |
|  | Model 2^d^ | 1 | 1.47 (1.25, 1.75) | | 1.28 (1.08, 1.52) | | 2.18 (1.86, 2.57) | | < 0.0001 | | 1.64 (1.47, 1.83) | | n/a | |
|  | Model 3^e^ | 1 | 1.48 (1.25, 1.75) | | 1.25 (1.05, 1.49) | | 2.19 (1.86, 2.58) | | < 0.0001 | | 1.63 (1.46, 1.82) | | n/a | |
|  |  |  |  | |  | |  | |  | |  | |  | |
| American black women | Model 1^c^ | 1 | 1.43 (1.11, 1.85) | | 1.82 (1.41, 2.36) | | 2.83 (2.18, 3.66) | | < 0.0001 | | 1.53 (1.35, 1.72) | | n/a | |
|  | Model 2^d^ | 1 | 1.47 (1.10, 1.96) | | 1.51 (1.14, 2.01) | | 2.37 (1.78, 3.15) | | < 0.0001 | | 1.46 (1.28, 1.67) | | n/a | |
|  | Model 3^e^ | 1 | 1.47 (1.10, 1.96) | | 1.50 (1.13, 2.00) | | 2.40 (1.79, 3.19) | | < 0.0001 | | 1.47 (1.28, 1.68) | | n/a | |
|  |  |  |  | |  | |  | |  | |  | |  | |
| **Hypertension** | |  |  | |  | |  | |  | |  | |  | |
| All participants | Model 1^c^ | 1 | 1.06 (0.94, 1.19) | | 1.43 (1.28, 1.61) | | 1.84 (1.64, 2.07) | | < 0.0001 | | 1.54 (1.43, 1.67) | | 0.002 | |
|  | Model 2^d^ | 1 | 0.99 (0.87, 1.12) | | 1.38 (1.22, 1.57) | | 1.56 (1.37, 1.77) | | < 0.0001 | | 1.40 (1.29, 1.53) | | 0.008 | |
|  | Model 3^e^ | 1 | 0.99 (0.87, 1.12) | | 1.37 (1.21, 1.56) | | 1.59 (1.40, 1.81) | | < 0.0001 | | 1.40 (1.29, 1.53) | | 0.005 | |
|  |  |  |  | |  | |  | |  | |  | |  | |
| American white women | Model 1^c^ | 1 | 1.07 (0.94, 1.22) | | 1.45 (1.28, 1.65) | | 1.86 (1.64, 2.12) | | < 0.0001 | | 1.73 (1.57, 1.92) | | n/a | |
|  | Model 2^d^ | 1 | 0.95 (0.83, 1.09) | | 1.41 (1.23, 1.62) | | 1.57 (1.37, 1.81) | | < 0.0001 | | 1.55 (1.39, 1.73) | | n/a | |
|  | Model 3^e^ | 1 | 0.96 (0.83, 1.10) | | 1.40 (1.21, 1.61) | | 1.61 (1.39, 1.86) | | < 0.0001 | | 1.56 (1.40, 1.74) | | n/a | |
|  |  |  |  | |  | |  | |  | |  | |  | |
| American black women | Model 1^c^ | 1 | 1.19 (0.92, 1.54) | | 1.36 (1.05, 1.76) | | 1.64 (1.26, 2.13) | | 0.0002 | | 1.21 (1.08, 1.36) | | n/a | |
|  | Model 2^d^ | 1 | 1.21 (0.91, 1.61) | | 1.31 (0.98, 1.75) | | 1.42 (1.06, 1.90) | | 0.025 | | 1.15 (0.999, 1.31) | | n/a | |
|  | Model 3^e^ | 1 | 1.16 (0.86, 1.55) | | 1.30 (0.97, 1.75) | | 1.38 (1.02, 1.85) | | 0.035 | | 1.13 (0.98, 1.30) | | n/a | |
|  |  |  |  | |  | |  | |  | |  | |  | |
| **CKD** |  |  |  | |  | |  | |  | |  | |  | |
| All participants | Model 1^c^ | 1 | 0.90 (0.67, 1.23) | | 1.28 (0.97, 1.70) | | 2.56 (1.98, 3.30) | | < 0.0001 | | 1.76 (1.59, 1.95) | | 0.773 | |
|  | Model 2^d^ | 1 | 0.74 (0.53, 1.04) | | 1.08 (0.79, 1.46) | | 2.29 (1.74, 3.01) | | < 0.0001 | | 1.84 (1.63, 2.08) | | 0.188 | |
|  | Model 3^e^ | 1 | 0.74 (0.53, 1.05) | | 1.03 (0.75, 1.39) | | 2.22 (1.68, 2.92) | | < 0.0001 | | 1.84 (1.63, 2.08) | | 0.176 | |
|  |  |  |  | |  | |  | |  | |  | |  | |
| American white women | Model 1^c^ | 1 | 0.82 (0.59, 1.14) | | 1.13 (0.83, 1.52) | | 2.29 (1.75, 3.00) | | < 0.0001 | | 1.83 (1.61, 2.09) | | n/a | |
|  | Model 2^d^ | 1 | 0.67 (0.47, 0.97) | | 0.93 (0.67, 1.29) | | 2.00 (1.50, 2.67) | | < 0.0001 | | 1.87 (1.62, 2.17) | | n/a | |
|  | Model 3^e^ | 1 | 0.68 (0.47, 0.97) | | 0.88 (0.63, 1.23) | | 1.93 (1.44, 2.59) | | < 0.0001 | | 1.88 (1.62, 2.17) | | n/a | |
|  |  |  |  | |  | |  | |  | |  | |  | |
| American black women | Model 1^c^ | 1 | 1.49 (0.60, 3.71) | | 2.48 (1.07, 5.74) | | 4.73 (2.14, 10.45) | | < 0.0001 | | 1.62 (1.39, 1.89) | | n/a | |
|  | Model 2^d^ | 1 | 1.60 (0.57, 4.45) | | 2.16 (0.83, 5.64) | | 4.22 (1.69, 10.53) | | 0.0002 | | 1.87 (1.51, 2.32) | | n/a | |
|  | Model 3^e^ | 1 | 1.58 (0.56, 4.40) | | 2.12 (0.81, 5.56) | | 3.95 (1.58, 9.88) | | 0.001 | | 1.89 (1.51, 2.36) | | n/a | |
|  |  |  |  | |  | |  | |  | |  | |  | |
| **Composite outcome (at least one comorbidity)** | | | |  | |  | |  | |  | |  | |  |
| All participants | Model 1^c^ | 1 | 1.16 (1.03, 1.30) | | 1.56 (1.39, 1.75) | | 2.57 (2.27, 2.91) | | < 0.0001 | | 2.13 (1.94, 2.34) | | 0.454 | |
|  | Model 2^d^ | 1 | 1.09 (0.96, 1.23) | | 1.56 (1.37, 1.77) | | 2.31 (2.03, 2.64) | | < 0.0001 | | 2.01 (1.82, 2.22) | | 0.218 | |
|  | Model 3^e^ | 1 | 1.09 (0.96, 1.23) | | 1.55 (1.36, 1.76) | | 2.35 (2.05, 2.68) | | < 0.0001 | | 2.01 (1.82, 2.22) | | 0.175 | |
|  |  |  |  | |  | |  | |  | |  | |  | |
| American white women | Model 1^c^ | 1 | 1.24 (1.10, 1.41) | | 1.53 (1.35, 1.74) | | 2.52 (2.21, 2.88) | | < 0.0001 | | 2.32 (2.07, 2.60) | | n/a | |
|  | Model 2^d^ | 1 | 1.16 (1.01, 1.32) | | 1.52 (1.33, 1.74) | | 2.33 (2.02, 2.69) | | < 0.0001 | | 2.22 (1.97, 2.50) | | n/a | |
|  | Model 3^e^ | 1 | 1.17 (1.02, 1.34) | | 1.50 (1.31, 1.73) | | 2.38 (2.06, 2.74) | | < 0.0001 | | 2.23 (1.98, 2.52) | | n/a | |
|  |  |  |  | |  | |  | |  | |  | |  | |
| American black women | Model 1^c^ | 1 | 1.25 (0.94, 1.65) | | 1.96 (1.44, 2.66) | | 2.28 (1.67, 3.12) | | < 0.0001 | | 1.74 (1.46, 2.07) | | n/a | |
|  | Model 2^d^ | 1 | 1.26 (0.92, 1.73) | | 1.64 (1.18, 2.29) | | 2.02 (1.44, 2.83) | | < 0.0001 | | 1.59 (1.33, 1.91) | | n/a | |
|  | Model 3^e^ | 1 | 1.22 (0.89, 1.68) | | 1.62 (1.16, 2.26) | | 1.96 (1.40, 2.76) | | < 0.0001 | | 1.58 (1.31, 1.90) | | n/a | |

^a^ ORs represent per-standard deviation increases in biomarker measures.

^b^ P for interaction was obtained by adding an interaction term between each vitamin D biomarker and race/ethnicity into each model; n/a indicates that interaction tests for race/ethnicity were not applicable.

^c^ Model 1 adjusted for age, clinical center, and race/ethnicity.

^d^ Model 2 further adjusted for BMI (for prevalent CKD and hypertension only), family history of CVD, educational levels, alcohol intake, physical activity levels, cigarette smoking status, postmenopausal hormone therapy use, and season of blood draw.

^e^ Model 3 additionally adjusted for eGFR (for prevalent obesity and hypertension only), history of high cholesterol, and statin use.
